# Relationship Between Impulsivity, Sensation-Seeking, and Drug Use in Aggressors and Victims of Violence

**DOI:** 10.3389/fpsyg.2020.600055

**Published:** 2020-10-30

**Authors:** María del Mar Molero Jurado, María del Carmen Pérez-Fuentes, María del Mar Simón Márquez, Ana Belén Barragán Martín, Maria Sisto, José Jesús Gázquez Linares

**Affiliations:** ^1^Department of Psychology, Faculty of Psychology, University of Almería, Almería, Spain; ^2^Department of Psychology, Faculty of Psychology, Universidad Politécnica y Artística del Paraguay, Asunción, Paraguay; ^3^Department of Psychology, Faculty of Psychology, Universidad Autónoma de Chile, Santiago, Chile

**Keywords:** drug, impulsivity, sensation-seeking, peer conflict, adolescent

## Abstract

**Introduction:** The impulsivity, sensation-seeking, and drug use variables, in addition to being closely related, have repercussions on peer conflict and violence in schools.

**Objective:** The purpose of this study was to analyze the relationship between impulsivity, sensation-seeking, and drug use in aggressors and victims of violence.

**Methods:** The study design was cross-sectional and observational. The study sample was made up of 822 students aged 13–18 who had completed an *ad hoc* questionnaire, the State Impulsivity Scale and the Sensation-Seeking Scale.

**Results:** The results showed that the aggressors had high levels of gratification, automatism, attentional factor, disinhibition, and susceptibility to boredom and used alcohol and/or tobacco.

**Conclusion:** The design of an effective education intervention for reducing risk behaviors related to violence must focus on these variables.

## Introduction

For years, peer violence, understood as a type of repeated intentional aggression toward another person with whom there is an imbalance of power and which may be physical or verbal and can occur in interpersonal relationships (Olweus, 1993), has been one of the most formidable problems in the school environment, although we cannot forget the increase in violence from Child-to-parent Violence and Parent-to-child Violence (Gallego et al., 2019). Peer violence may increase when students suffer from problems of anxiety or depression at the beginning of the school year (Fekkes et al., 2006), which could give them the appearance of being vulnerable and make them easier targets for potential aggressors (Crick et al., 1999).

This phenomenon has many causes (Bohnert et al., 2003; Hemphill et al., 2012; Jiménez and Estévez, 2017) and likewise, predisposing factors have been found which would increase the probability of a person becoming involved in school violence (Romero-Abrio et al., 2019). Among some of the most important are low levels of emotional skills in aggressors (Inglés et al., 2014), family characteristics, such as lack of care, supervision, control, and parental affect (Dracic, 2009; Cutrín et al., 2018; Rodríguez et al., 2018; Pérez-Fuentes et al., 2019a), and substance use (Bender and Lösel, 2011; Cerezo and Méndez, 2013; Pérez-Fuentes et al., 2019b).

Adolescence is a critical period for developing disorders related to substance abuse (Verdejo-García et al., 2008), as reflected in the State Survey on High School Drug Use, ESTUDES 2016–2017 (Ministry of Health Social Services and Equality, 2018), which shows that alcohol and tobacco continue to be the drugs most used by students from 14 to 18 years old. In addition to these figures, the risk of developing disorders related to alcohol abuse is twice as high for adolescents as for young adults and seven times higher for adolescents aged 15–16 compared to young adults 22–26 in the case of disorders related to cannabis (Winters and Lee, 2008).

Drug use has a more complex relationship than simply a tendency for the appearance of bullying. A stronger tendency toward substance use and abuse has been found in young people who were aggressors during adolescence (Moore et al., 2014). Paradoxically, this is also the case of victims, whose probability of using substances increases (Romaní and Gutierrez, 2010). In this respect, an apparently contradictory tendency found is that adolescents who smoke cigarettes, use marihuana, or drink alcohol have a lower probability of being victims of bullying (García et al., 2008). Such results show the obvious relationship between substance use and problematic, maladaptive, or risk behavior (Otero, 2001).

Impulsivity, a trait which has also been described as a factor predisposing a person to participation in violence, characterizes aggressors, or bullies (Pichardo et al., 2005) and is associated with other behaviors, such as substance use, for which high school students who said they had drunk alcohol or smoked at some time, or did so habitually, scored significantly higher (Pérez-Fuentes et al., 2015a). Dysfunctional impulsivity (Dickman, 1990) has specifically been shown to be a construct with a close relationship to addiction (Pedrero, 2009). Preliminary studies have analyzed whether this is the cause or the consequence of substance use and abuse behaviors, a matter mentioned above, and the results seem to indicate that, in the aggressor or bully profile, substance use promotes aggressive behavior toward others (Kaltiala-Heino et al., 2000; Moñino et al., 2013) and is also related to school failure. This, in turn, is a vulnerability factor for substance use and polyuse, as well as for antisocial behaviors (Cerezo and Méndez, 2012).

Sensation-seeking as a personality trait is the search for new, different, complex, and intense sensations and experiences and the tendency for behavior which places physical health and social, legal, and economic situations at risk to achieve the satisfaction of such experiences (Zuckerman, 1994). Impulsivity and sensation-seeking are some of the personality factors relevant to the appearance of risk behaviors (Rosenbloom, 2003). Sensation-seeking also has an important role in the dynamics of bullying, such as substance use. In the 1980s, it was found that excessive alcohol intake was strongly related to sensation-seeking, resulting in a more obvious relationship for abuse than for use (Cárdenas and Moreno-Jiménez, 1989). This has been replicated in more recent research (Eskandari and Helmi, 2014; Leeman et al., 2014). Furthermore, Kirsh (2003) found that subjects with high sensation-seeking traits felt more attracted by violent images, which has also been associated with different forms of aggression and the possibility of becoming involved in cyberbullying (Kokkinos et al., 2015). Such data, despite showing the relationship between sensation-seeking and violent behaviors, are insufficiently specific to come to conclusions on whether this construct can predict such violent behavior among peers, and this is one of the objectives that this study intended to achieve.

The aggressors have certain characteristics, among which the most important are impulsivity (Low and Espelage, 2014), hyperactive behavior patterns (Nabuzoka, 2003), low academic performance, often leading to the student repeating a year (Pérez-Fuentes et al., 2015b), low levels of benevolence (Gázquez et al., 2015), and a strong tendency to be distracted and limited prosocial interaction (Cho et al., 2009). A tendency to limited prosocial interaction has a negative influence on academic performance, as it has been found that high school students with high scores in prosocial behavior tend to show a positive attitude, motivation, exam preparation strategies, etc. (Inglés et al., 2013; Gázquez et al., 2016).

On the contrary, the victims usually have a lower level of self-esteem (Malti et al., 2010; Garaigordobil et al., 2013), specific needs for educational support (Martos and Del Rey, 2013), feelings of unhappiness, rejection by their peer group, anxiety, and depression (Rigby, 2003), which can lead to students having depressive episodes (Jaureguizar et al., 2015). When coping with bullying, victims are characterized by trying to get out of the situation repeatedly, and in different ways, but unsuccessfully, which seems to indicate that they do not develop adequate resources that would enable them to end the abuse (Rodríguez and Mora-Merchán, 2014). The impulsivity, sensation-seeking, and drug use variables have been found to be closely related. They, in turn, also have a close relationship with peer conflict. The purpose of this study was to analyze the relationship between impulsivity, sensation-seeking and drug use in aggressors and victims of violence.

Based on prior empirical evidence, the following hypotheses were posed: (1) aggressors have higher scores in traits associated with impulsivity and sensation-seeking than non-aggressors, (2) victims show differences from non-victims in their lower impulsivity and sensation-seeking scores, and (3) impulsivity traits, the tendency to sensation-seeking, and drug and tobacco use are predictors of involvement of the participants in episodes of peer violence.

## Materials and Methods

### Participants

The sample was taken by random cluster sampling by the geographic zones into which the city of Almeria (Spain) is divided, for which eight high schools were selected at random. The size of the starting sample was set at 906, randomly selected for a 3.2% confidence interval, with a 95% confidence level and a 0.5 population variance. Twenty-three questionnaires were disqualified because of a systematically inconsistent response pattern for the questions asked, and 61 were discarded because they were incomplete. Thus, the final sample consisted of 822 students from the 3rd and 4th year of high school, with an age range of 13–18 and a mean of 14.84 years (SD = 0.87). Of the total sample, 51.8% (*n* = 426) were male and 48.2% (*n* = 396) were female, with mean ages of 14.85 (SD = 0.87) and 14.82 years (SD = 0.86), respectively. The grade distribution of the sample was as follows: 43.7% were in 3rd year (*n* = 359) and 56.3% were in 4th year (*n* = 463).

### Instruments

A questionnaire to find out the sociodemographic characteristics of the participants, whether they were an aggressor (Have you ever used/do you use violence against your fellow students?) or a victim (Have you ever been the object of violence by your fellow students?) (Pérez-Fuentes et al., 2015b), and items in which they were asked about their alcohol drinking (How often do you drink alcoholic beverages (glasses/drinks)?) and tobacco smoking (How often do you smoke cigarettes?) (Delegación del Gobierno para el Plan Nacional sobre Drogas, 2016).

The State Impulsivity Scale by Iribarren et al. (2011) as designed to evaluate impulsive behavior defined as a state, that is impulsivity as a manifest behavior which can vary in the short-term. It consists of 20 items, distributed into three subscales: gratification (evaluates the urgency in satisfying impulses, preference for immediate reward, intolerance of frustration, and the tendency to act without thinking about any negative consequences), automatism (refers to behavior expressed rigidly and repeatedly, without paying attention to contextual variables), and attentional (evaluates the presence of unplanned behaviors which take place because of having acted too soon and without considering all the information available). The response is based on a four-point Likert scale where the participants are asked to evaluate the frequency with which each of the statements applies to them. The authors (Iribarren et al., 2011) found high reliability for both the total scale (α = 0.88) and each of its dimensions: gratification (α = 0.84), automatism (α = 0.80), and attentional (α = 0.75).

The Sensation-Seeking Scale was also used (Pérez and Torrubia, 1986). Its 40 yes/no response items evaluate the trait for seeking new, risky experiences. It comprises four subscales: emotion-seeking (EMS), excitement-seeking (EXS), disinhibition (DIS), and susceptibility to boredom (STB).

### Procedure

The study design was cross-sectional and observational. First, the principals of each school were informed of the objectives, procedure, and use of the data for research. In addition, parents/guardians were asked for the pertinent permission on an informed consent sheet. Before the tests were administered, the participants were given instructions on how to complete them and were guaranteed the confidentiality of data processing. Then, two members of the research team went to the schools to administer the standardized anonymous self-report questionnaire (paper and pencil) to all the participants in a single 40–45-min session. The study was approved by the Bioethics Committee of University of Almería (Ref. UALBIO2018/015).

### Data Analysis

For the use variables (alcohol/tobacco), it was unnecessary to set a threshold, since grouping was by whether this characteristic was given or not. However, the criteria for defining the sample thresholds (high and low) for the interpersonal value scales were identified, and the normal distribution on the State Impulsivity and Sensation-Seeking questionnaires was tested. The total sample was divided into two groups for each of the scales based on the gratification, automatism, and attentional scores: (a) subjects with low scores, who scored the same or lower than the 25th percentile (scores equal to or higher than 10, 9, and 11, respectively), and (b) subjects with high scores, who scored the same or higher than the 75th percentile (scores the same or higher than 16, 14, and 17, respectively). Two groups were formed for sensation-seeking on the emotion-seeking, excitement-seeking, disinhibition, and susceptibility to boredom scales: (a) subjects with low scores who scored the same or lower than the 25th percentile (scores the same or higher than 4, 4, 3, and 3, respectively), and (b) subjects with high scores who scored the same or higher than the 75th percentile (scores the same or higher than 8, 6, 6, and 6, respectively).

Bivariate analysis with Chi-square was used for participant differences. To test the predictive power of trait impulsivity and sensation-seeking for determining aggressors or non-aggressors and victims or non-victims, univariate logistic regression models and bivariate and multivariate forward stepwise logistic regression were applied using the Wald test with the criterion variables (aggressor and victim) and the nine predictor variables (alcohol use, tobacco use, gratification, automatism, attentional, emotion-seeking, excitement-seeking, disinhibition, and susceptibility to boredom). Then a nonlinear predictive Chi-Square Automatic Interaction Detector (CHAID) regression and classification tree was constructed for aggressors or non-aggressors.

Finally, a simple mediation analysis was performed for alcohol and tobacco, taking involvement as the aggressor in school violence as the dependent variable. The SPSS macro for mediation models was used for this (Hayes, 2013). Bootstrapping was performed with coefficients estimated from 5000 bootstraps.

## Results

Table 1 shows the sample distribution with the differences between aggressor (*n* = 82) and non-aggressor (*n* = 740) groups. There was a higher presence of aggressors among those with high gratification (17.1%), high automatism (14.6%), and attentional (15%) compared to low levels (5.2, 6.3, and 5.1%, respectively), differences which were significant (*p* < 0.01) in all cases. Furthermore, in the analysis of the sensation-seeking dimensions, there was a higher prevalence of aggressors among participants who had high disinhibition (17.5%) and susceptibility to boredom (13%), with significant differences (*p* < 0.01) from low scores (4.2 and 5.9%, respectively).

**TABLE 1:** Aggressor/non-aggressor percentages.

		Non-aggressor (%)	Aggressor (%)	χ^2^	*p*
Use alcohol	No	92.7	7.3	14.38	0.00
	Yes	84.1	15.9		
Use tobacco	No	94.1	5.9	7.26	0.01
	Yes	88.2	11.8		
GRA	Low	94.8	5.2	16.82	0.00
	High	82.9	17.1		
AUTO	Low	93.7	6.3	9.86	0.01
	High	85.4	14.6		
ATEN	Low	94.9	5.1	13.61	0.00
	High	85	15		
EMS	Low	90.4	9.6	0.25	0.61
	High	89	11		
EXS	Low	92.1	7.9	1.39	0.24
	High	92.1	7.9		
DIS	Low	95.8	4.2	25.69	0.00
	High	82.5	17.5		
STB	Low	94.1	5.9	7.78	0.01
	High	87	13		

*GRA, gratification; AUTO, automatism; ATEN, attentional; EMS, emotion-seeking; EXS, excitement-seeking; DIS, disinhibition; STB, susceptibility to boredom.*

A majority frequency of aggressors who use alcohol (15.9%) and tobacco (11.8%) was observed, with statistically significant differences (*p* < 0.01) in both cases.

The same analysis completed for victims (*n* = 88) and non-victims (*n* = 732) (Table 2) shows that even though the differences are not statistically significant a higher prevalence of victims may be observed among those who have low levels of gratification (11.3%), attentional (9.3%), disinhibition (12.1%), and susceptibility to boredom (10.5%) and a higher frequency of victims among those who have high scores in automatism (13.4%), emotion-seeking (10.7%), and excitement-seeking (11%). No significant differences were observed in the frequency of victims/non-victims with alcohol or tobacco.

**TABLE 2:** Percentages of victims/non-victims.

		Non-victim (%)	Victim (%)	χ^2^	*p*
Use alcohol	No	90.1	9.9	1.38	0.24
	Yes	87.3	12.7		
Use tobacco	No	89	11	0.03	0.85
	Yes	89.4	10.6		
GRA	Low	88.7	11.3	0.06	0.81
	High	89.4	10.6		
AUTO	Low	91.5	8.5	3.26	0.07
	High	86.6	13.4		
ATEN	Low	90.7	9.3	0.02	0.89
	High	91	9		
EMS	Low	89.5	10.5	0.01	0.96
	High	89.3	10.7		
EXS	Low	91	9	0.63	0.43
	High	89	11		
DIS	Low	87.9	12.1	1.25	0.26
	High	90.9	9.1		
STB	Low	89.5	10.5	0.02	0.90
	High	89.9	10.1		

*GRA, gratification; AUTO, automatism; ATEN, attentional; EMS, emotion-seeking; EXS, excitement-seeking; DIS, disinhibition; STB, susceptibility to boredom.*

As shown in Table 3, the variables which can predict involvement as an aggressor in peer conflict are drinking alcohol and smoking, with high levels in the gratification, automatism, and attentional factors, as well as high disinhibition and susceptibility to boredom.

**TABLE 3:** Univariate logistic regression for the probability of being an aggressor.

Total	*B*	SE	Wald	*p*	OR	CI	*R*^2^ Nagelkerke
Use alcohol	0.70	0.28	6.19	0.01	2.02	1.16–3.52	0.02
Use tobacco	0.85	0.24	13.14	0.00	2.35	1.48–3.73	0.03
GRA	1.31	0.34	15.15	0.00	3.72	1.92–7.20	0.07
AUTO	0.96	0.31	9.94	0.01	2.61	1.44–4.75	0.04
ATEN	1.19	0.34	12.44	0.00	3.30	1.70–6.41	0.06
DIS	1.59	0.34	21.97	0.00	4.88	2.52–9.48	0.10
STB	0.81	0.31	6.72	0.01	2.26	1.22–4.18	0.03

*GRA, gratification; AUTO, automatism; ATEN, attentional; EMS, emotion-seeking; EXS, excitement-seeking; DIS, disinhibition; STB, susceptibility to boredom; *B*, coefficient; SE, standard error; OR, odds ratio; CI, confidence interval at 95%.*

The aspects which showed an association with each final objective at *p* < 0.01 were included in the model in the univariate analysis. Thus, the fit by a multivariate logistic regression model which is the correct classification of 22.3% (*R*^2^ = 0.22) shows the high scores in disinhibition (OR = 11.93; 95% CI = 2.47, 57.65) as a variable associated with the aggressor profile (Table 4).

**TABLE 4:** Logistic regression for the probability of being an aggressor.

Total	*B*	SE	Wald	*p*	OR	CI	*R*^2^ Nagelkerke
DIS	2.48	0.80	9.52	0.01	11.94	2.47–57.66	0.22

*DIS, disinhibition; *B*, coefficient; SE, standard error; OR, odds ratio; CI, confidence interval at 95%.*

**FIGURE 1:**
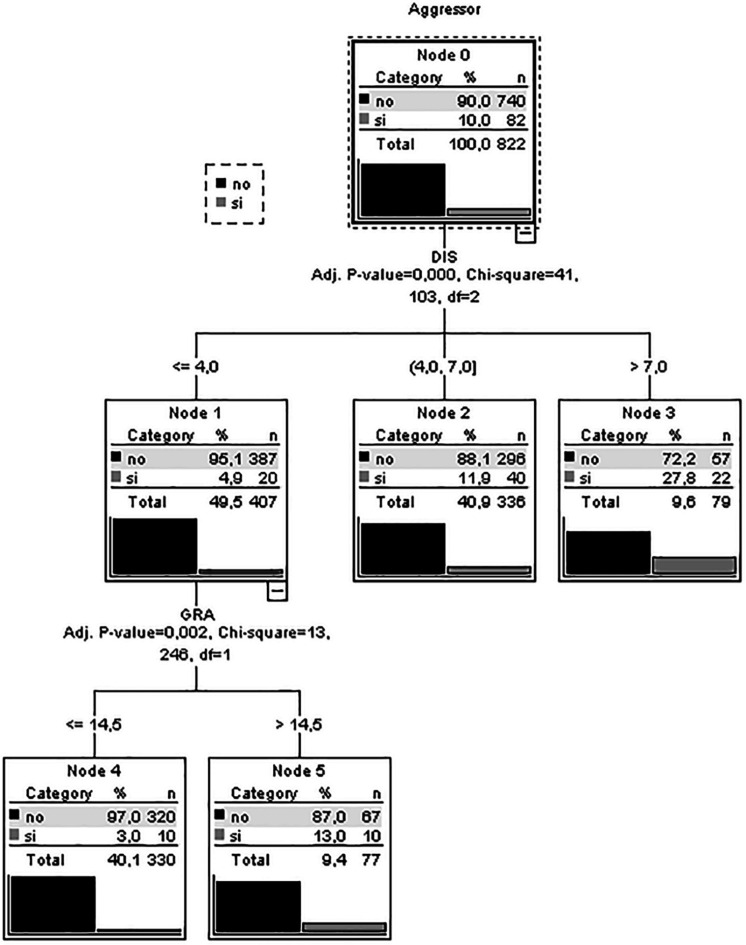
Decision tree analysis.

As observed in the decision tree (Figure 1), disinhibition is the best predictor of an aggressor. Participants with lower scores in disinhibition and gratification have a lower probability of being an aggressor (97%). The greatest risk of being an aggressor (27.8%) is among those who have higher scores in disinhibition. A good fit of model functioning can be observed in its correct classification of 90% of the participants.

A simple mediation model for use of alcohol and another for tobacco were computed based on the results of the regression analyses estimated, in which the mediating variable was disinhibition in both cases, to predict involvement as the aggressor in peer violence.

**FIGURE 2:**
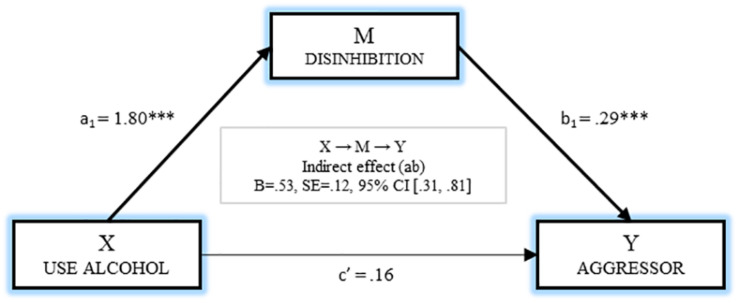
Mediation of disinhibition on the relationship between use alcohol and being aggressor.

Figure 2 shows the simple mediation model for analyzing the mediating effect of disinhibition on the relationship between drinking alcohol and being an aggressor. In the first regression analysis, disinhibition was taken as the result variable (M), and the effect of drinking alcohol was estimated, and found to be significant [*B* = 1.80, *p* < 0.001]. With the next regression analysis, taking involvement as the aggressor as the result variable (Y), the effect of the independent variable (*B* = 0.16, *p* = 0.592) and the mediator (*B* = 0.29, *p* < 0.001) were estimated, and only one significant M→Y effect was observed. Furthermore, the analysis of the indirect effects by bootstrapping found a significant effect [*B* = 0.53, SE = 0.125, 95% CI (0.312, 0.811)].

**FIGURE 3:**
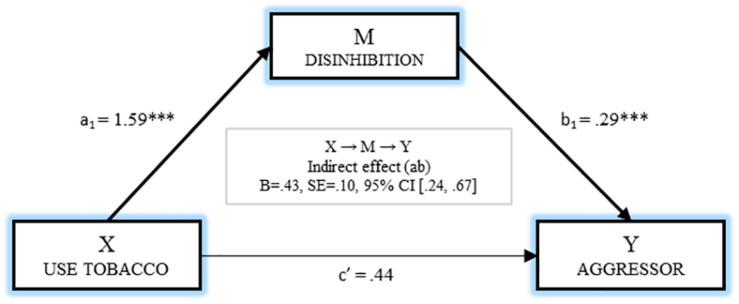
Mediation of disinhibition on the relationship between use tobacco and being aggressor.

The analysis of the mediating effect of disinhibition on the relationship between smoking and being the aggressor is shown in Figure 3. It may be observed that the effect of using tobacco on disinhibition was significant (*B* = 1.59, *p* < 0.001). The effect of the independent variable (*B* = 0.44, *p* = 0.080) and the mediator (*B* = 0.29, *p* < 0.001) on becoming the aggressor (Y) was estimated by the next regression analysis, and a significant M→Y effect was observed. Finally, bootstrapping extracted data supporting a significant indirect effect [B = 0.43, SE = 0.107, 95% CI (0.249, 0.675)].

No multivariate model analysis was performed for the victim profile, as no association was found for any of the variables in the univariate analysis at *p* < 0.01 (Table 5).

**TABLE 5:** Univariate logistic regression for the probability of being a victim.

Total	*B*	SE	Wald	*p*	OR	CI	*R*^2^ Nagelkerke
Use alcohol	–0.04	0.23	0.03	0.85	0.95	0.60–1.52	0.00
Use tobacco	0.27	0.23	1.37	0.24	1.32	0.83–2.10	0.00
GRA	–0.07	0.29	0.05	0.81	0.93	0.51–1.67	0.00
AUTO	0.50	0.28	3.21	0.07	1.66	0.95–2.90	0.01
ATEN	–0.04	0.31	0.02	0.88	0.95	0.51–1.76	0.00
EMS	0.01	0.28	0.00	0.95	1.01	0.58–1.77	0.00
EXS	0.21	0.27	0.63	0.42	1.24	0.72–2.11	0.00
DIS	–0.31	0.28	1.24	0.26	0.72	0.41–1.27	0.00
STB	–0.03	0.29	0.01	0.89	0.96	0.53–1.72	0.00

*GRA, gratification; AUTO, automatism; ATEN, attentional; EMS, emotion-seeking; EXS, excitement-seeking; DIS, disinhibition; STB, susceptibility to boredom. *B*, coefficient; SE, standard error; OR, odd ratio; CI, confidence interval at 95%.*

## Discussion

First, based on the data from the frequency analysis, a higher percentage of aggressors were found among the subjects, who showed high levels of the gratification, automatism, and attentional factors. Furthermore, a higher frequency of aggressors was observed among the participants in the sample who had higher scores in disinhibition and susceptibility to boredom. In addition, a larger number of aggressors were also found among those who used alcohol and/or tobacco. These initial results already provide clues to the relationship of impulsivity traits (Low and Espelage, 2014), the tendency for sensation-seeking (Kirsh, 2003) and substance use (Bender and Lösel, 2011; Cerezo and Méndez, 2013) to the aggressor profile.

School violence victims showed a contrary tendency to the one observed in aggressors; although there were no significant differences in the percentages with respect to non-victims, a larger number of victims among the participants had low levels of gratification, attentional, disinhibition, and susceptibility to boredom. Results such as these suggest the different forms of coping which aggressors and victims use in a conflict situation (Rodríguez and Mora-Merchán, 2014). The literature refers to the difficulty victims have in developing and/or initiating effective resources, which leads to negative effects associated with the coexistence profile they adopt (Malti et al., 2010; Garaigordobil et al., 2013; Martos and Del Rey, 2013; Jaureguizar et al., 2015).

Disinhibition is the best predictor of an aggressor. The logistic regression analysis provided additional empirical evidence in support of the association of certain impulsivity and sensation-seeking traits and alcohol and tobacco use and their predictive value for involvement in aggressive behaviors with peers at school. In addition, it was observed from the mediation models computed, that disinhibition exerted an important mediating role in the relationship between use (alcohol/tobacco) and acting as the aggressor in school violence. In this case, using alcohol or tobacco did not have a significant direct effect on becoming an aggressor. However, the use variables did acquire relevance when the mediating effect of disinhibition was included. With regard to these results, the importance of performing an analysis of this type, which reveals data supporting the need to examine the position of each of the variables as part of a model, should be mentioned.

Specifically, the tendency for impulsive behavior (with high levels in the gratification, automatism, and attentional factors) has already been proposed as a factor which predisposes to active participation in episodes of violence (Pichardo et al., 2005). Substance use, which is closely related to impulsivity (Pedrero, 2009; Pérez-Fuentes et al., 2015a), is another of the risk factors that can predict violent behavior by adolescents (Kaltiala-Heino et al., 2000; Moñino et al., 2013; Pérez-Fuentes et al., 2019b) and is also a later consequence of having adopted an aggressive profile during adolescence (Moore et al., 2014). Finally, sensation-seeking, especially in combination with impulsivity, has an important role in explaining and/or predicting risk behavior, such as substance use (Rosenbloom, 2003; Eskandari and Helmi, 2014; Leeman et al., 2014), or involvement in bullying at school (Kokkinos et al., 2015).

One of the limitations of this work is that we cannot establish causal relationships, as we simply analyzed the relationship of the variables. Therefore, future research designs should enable analysis of the causal theory of the severity of victimization. Furthermore, since we cannot establish causal relationships, a disinhibition intervention program should be implemented to find out whether the aggressive behavior of the subject really diminishes. This could be the goal of another future study.

## Conclusion

The variables that could predict involvement as an aggressor in peer conflict are use of alcohol, smoking, high levels of the gratification, automatism, and attentional factors, and a high degree of disinhibition and susceptibility to boredom. Thus, having empirical evidence available which facilitates the detection of predictive variables of involvement in acts of violence, with disinhibition being the best predictor of an aggressor, is going to enable the design of effective interventions for reducing risk behaviors. At the same time, this evidence will acknowledge the importance of working to acquire coping skills and/or strategies in adolescent conflict situations as an alternative to the use of violence.

In view of the results, a positive approach must be adopted for treating adolescent risk behaviors such as direct involvement in peer violence. In this process of change, the education community has a fundamental role, and needs to know the variables involved in the design of specific action in this context and be able to identify the presence of risk indicators. Therefore, by focusing on identifying the variables most predictive of violent behavior, the educational patterns most coherent with the characteristics of this population group can be anticipated, the context for analysis and application evaluated, and resources provided to parents, educators, orientation teams, and even the students themselves.

## Data Availability Statement

The data that support the findings of this study are available from the corresponding author upon reasonable request.

## Ethics Statement

The studies involving human participants were reviewed and approved by the University of Almería (Ref. UALBIO2018/015). Written informed consent to participate in this study was provided by the participants’ legal guardian/next of kin.

## Author Contributions

MP-F, MMJ, MSM, and JG contributed to the conception and design of the review. JG applied the search strategy. MMJ, MP-F, and MSM wrote the manuscript. MMJ, MP-F, AB, and MS edited the manuscript. MP-F was responsible for the overall project. All authors applied the selection criteria, completed the assessment of risk of bias, and analyzed and interpreted the data.

## Conflict of Interest

The authors declare that the research was conducted in the absence of any commercial or financial relationships that could be construed as a potential conflict of interest.
